# Development and first results of a national databank on care and treatment outcome after traumatic brain injury

**DOI:** 10.1007/s00068-023-02260-6

**Published:** 2023-04-06

**Authors:** Alexander Younsi, Andreas Unterberg, Ingo Marzi, Wolf-Ingo Steudel, Eberhard Uhl, Johannes Lemcke, Florian Berg, Mathias Woschek, Michaela Friedrich, Hans Clusmann, Hussam Aldin Hamou, Uwe Max Mauer, Magnus Scheer, Jürgen Meixensberger, Dirk Lindner, Kirsten Schmieder, Mortimer Gierthmuehlen, Christine Hoefer, Ulrike Nienaber, Marc Maegele, for The TBI Databank Expert Group, Stefan Wolf, Bedjan Behmanesh, Ralf Watzlawick, Michael Bender, Hans-Peter Howaldt, Veit Rohde, Levent Tanrikulu, Patrick Czorlich, Pedram Emami, Florian Wild, Majid Esmaeilzadeh, Anna Prajsnar-Borak, Raimund Firsching, Michael Luchtmann, Markus Holling, Martin Strowitzki, Felix Reuter, Miron Yousif, Rolf Lefering, Thomas Westermaier, Christian Stetter, Björn Sommer, Yannik Bullinger

**Affiliations:** 1 Neurochirurgische Klinik, Universitätsklinikum Heidelberg, INF 400, 69120 Heidelberg, Germany; 2 Klinik für Unfall-, Hand- und Wiederherstellungschirurgie, Universitätsklinikum, Johann Wolfgang-Goethe-Universität, Frankfurt am Main, Germany; 3 Universitätsklinikum des Saarlandes, Homburg, Saar Germany; 4 Neurochirurgische Klinik, Universitätsklinikum Gießen und Marburg Standort Gießen, Giessen, Germany; 5 Klinik für Neurochirurgie, BG Klinikum Unfallkrankenhaus Berlin, Warener Straße 7, 12683 Berlin, Germany; 6 Klinik für Neurochirurgie, Uniklinik RWTH Aachen, Aachen, Germany; 7 Neurochirurgische Klinik, Bundeswehrkrankenhaus Ulm, Ulm, Germany; 8 Klinik und Poliklinik für Neurochirurgie, Universitätsklinikum Leipzig, Leipzig, Germany; 9 Universitätsklinikum Knappschaftskrankenhaus Bochum GmbH, Ruhr - Universität Bochum, In Der Schornau 23-35, 44892 Bochum, Germany; 10 Akademie der Unfallchirurgie GmbH, Emil-Riedel-Straße 5, 80538 Munich, Germany; 11 Klinik für Orthopädie, Unfallchirurgie und Sporttraumatologie, Klinikum Köln-Merheim, Institut für Forschung in der Operativen Medizin (IFOM), Universität Witten/Herdecke, Campus Köln-Merheim, Ostmerheimerstr. 200, 51109 Cologne, Germany

**Keywords:** Traumatic brain injury, Germany, Databank, Registry, Epidemiology, Outcome

## Abstract

**Purpose:**

In absence of comprehensive data collection on traumatic brain injury (TBI), the German Society for Neurosurgery (DGNC) and the German Society for Trauma Surgery (DGU) developed a TBI databank for German-speaking countries.

**Methods:**

From 2016 to 2020, the TBI databank DGNC/DGU was implemented as a module of the TraumaRegister (TR) DGU and tested in a 15-month pilot phase. Since its official launch in 2021, patients from the TR-DGU (intermediate or intensive care unit admission via shock room) with TBI (AIS head ≥ 1) can be enrolled. A data set of > 300 clinical, imaging, and laboratory variables, harmonized with other international TBI data collection structures is documented, and the treatment outcome is evaluated after 6- and 12 months.

**Results:**

For this analysis, 318 patients in the TBI databank could be included (median age 58 years; 71% men). Falls were the most common cause of injury (55%), and antithrombotic medication was frequent (28%). Severe or moderate TBI were only present in 55% of patients, while 45% suffered a mild injury. Nevertheless, intracranial pathologies were present in 95% of brain imaging with traumatic subarachnoid hemorrhages (76%) being the most common. Intracranial surgeries were performed in 42% of cases. In-hospital mortality after TBI was 21% and surviving patients could be discharged after a median hospital stay of 11 days. At the 6-and 12 months follow-up, a favorable outcome was achieved by 70% and 90% of the participating TBI patients, respectively. Compared to a European cohort of 2138 TBI patients treated in the ICU between 2014 and 2017, patients in the TBI databank were already older, frailer, fell more commonly at home.

**Conclusion:**

Within five years, the TBI databank DGNC/DGU of the TR-DGU could be established and is since then prospectively enrolling TBI patients in German-speaking countries. With its large and harmonized data set and a 12-month follow-up, the TBI databank is a unique project in Europe, already allowing comparisons to other data collection structures and indicating a demographic change towards older and frailer TBI patients in Germany.

## Introduction

Current German Federal Health Reporting System figures show that traumatic brain injury (TBI) remains a relevant clinical picture in Germany. For example, 421,060 patients with an injury to the head (ICD-10: S00-S09) were treated as full inpatients throughout Germany in 2019, an increase of 21% compared with 2000 [1]. As a result, the incidence of TBI in Europe is estimated at 47.3–849/100,000 inhabitants per year [2] and as high as 801-1299/100,000 inhabitants worldwide [3].

However, due to different data sources, documentation methods, and non-uniform definitions, only minimal general statements can be made about the epidemiology of TBI, even in Germany. Furthermore, disease-specific data on TBI are often only available based on individual studies. These current shortcomings are problematic in that detailed, robust, and area-wide data would be highly relevant for reviewing the clinical treatment of TBI patients, developing and implementing preventive measures, and assessing and quantifying the socioeconomic burden of TBI [4].

In Germany, TBI patients have been included in the trauma register (TR) of the German Society for Trauma Surgery (DGU) since 1993 as part of the acute care of severely injured patients. This TraumaRegister DGU^®^ (TR-DGU) is now carried out nationwide at approximately 630 certified hospitals/centers participating in trauma care and thus covers the whole country [5]. Since the dataset of the TR-DGU was not primarily designed to collect data on the care of TBI, it lacks the necessary scope of recorded variables and, thus, the corresponding specificity for a more detailed consideration of TBI [6].

Therefore, since 2016, within the framework of close cooperation between the DGU and the German Society for Neurosurgery (DGNC), the consolidation of the existing data collection structure of the TR-DGU with a data set specific and standardized for TBI has been advanced. In the following, the development process of this now comprehensive TBI databank DGNC/DGU of the TraumaRegister DGU (in short: TBI databank) is described, its first results are presented and compared to a large European TBI cohort.

## Methods

### Interdisciplinary approach

**Fig. 1:**
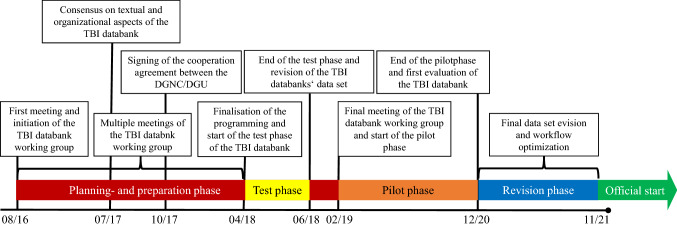
Timeline for the development and implementation of the TBI databank

After intensive preparatory work by a group of experts from traumatology and neurosurgery, a cooperation agreement between the DGNC and DGU was signed in October 2017 (Fig. 1). In this agreement, the goals for the planned databank included a review/improvement of the care of severely injured patients with TBI and registry-based quality assurance. For this purpose, it was decided to develop the TBI databank as an additional module of the nationally implemented TR-DGU.

**Fig. 2:**
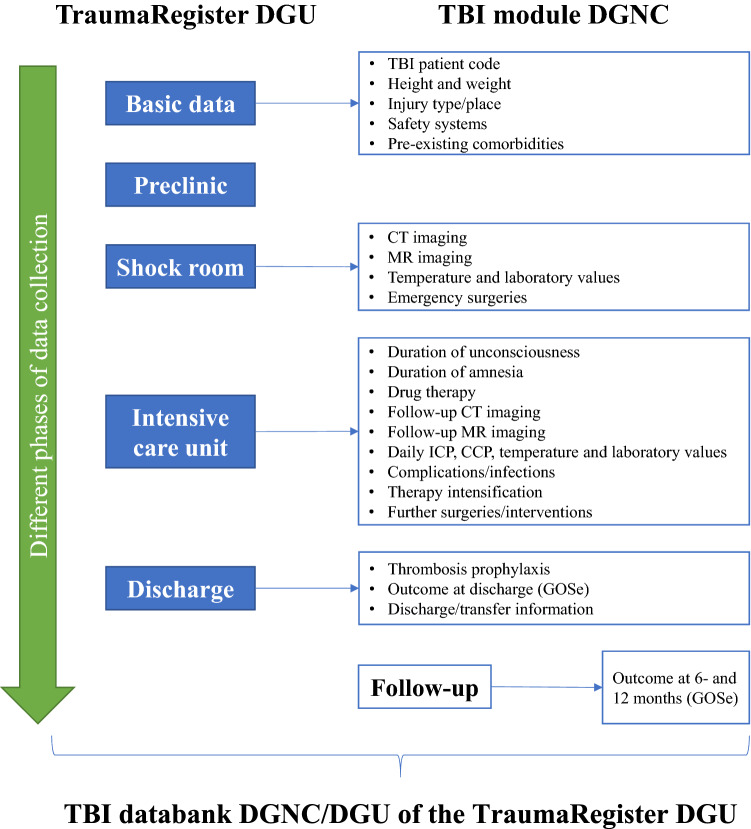
Different phases of the data collection in the TBI databank

Data collection in the TR-DGU is standardized and prospective, divided into master data and four acute care phases (Fig. 2) [5]. Inpatient admission via shock room with subsequent monitoring in the intermediate care unit (IMC) or intensive care unit (ICU) act as the inclusion criteria. However, severely injured patients already dying in the emergency department are also included. The technical development and implementation of the TBI databank were carried out by the AUC—Academy for Trauma Surgery (AUC—Akademie der Unfallchirurgie GmbH) (AUC), which is already responsible for the infrastructure of the TR-DGU and is scientifically accompanied by the Emergency, Intensive Care, and Serious Injury Care (NIS) section of the DGU.

### Dataset development and consensus

The focus for the variables to be recorded in the TBI databank was on comparability with other international TBI data collection structures. These, in turn, are based on “common data elements” standardized for TBI [7, 8]. The expert group initially developed a preliminary dataset with 483 variables on master data, prehospital, shock room/surgery phase, intensive care unit, and discharge/treatment outcome. Next, the newly developed dataset was tested in a three-month test phase at two neurosurgical and trauma surgery hospitals, followed by a revision that reduced the total number of variables for the pilot phase of the TBI databank to 327. The final dataset of the TBI databank, further refined and reduced after its pilot phase, captures information from demographics, clinic, imaging, shock room and ICU treatment, laboratory diagnostics, complications, and treatment outcomes (Fig. 2).

### Pilot phase and finalization of the TBI databank

**Fig. 3:**
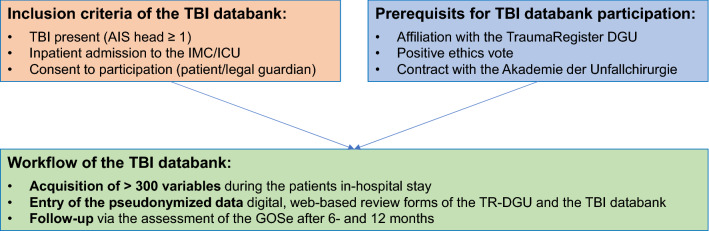
Inclusion criteria, prerequisites for participation and workflow of the TBI databank

For the pilot phase of the TBI databank, seven neurosurgical and two trauma surgery hospitals already members of the TraumaNetwork DGU were enabled by the AUC for data entry between 01/2019 and 12/2020. For this purpose, a contractual “Supplementary Agreement TBI Module” had to be concluded with the AUC. Furthermore, a positive ethics vote of the local ethics committee was necessary for patient inclusion. After completion of the pilot phase, revisions to the data set were made and problems with the software and the enrollment process eliminated. Since November 2021, the TBI databank is open to all TR-DGU-associated hospitals in German-speaking countries. Patients can be entered into the TBI databank if the above inclusion criteria for the TR-DGU are fulfilled and, in addition, if they have a TBI defined by at least a minor injury to the head (Abbreviated Injury Scale (AIS) Head Code ≥ 1) and if they or a legal guardian consented to participation (Fig. 3). Collected patient data are documented pseudonymously in digital review forms of the TR-DGU and the TBI databank via separate, web-based platforms of the AUC. Follow-up is performed at 6 and 12 months and consists of an assessment of the Glasgow Outcome Scale Extended (GOSe), an 8-item scale of recovery after TBI that dichotomizes treatment outcome into unfavorable (GOSe 1–4) and favorable (GOSe 5–8) [9].

### External benchmark: CENTER-TBI

To demonstrate the capabilities of the TBI databank’s data set, its results were compared to previously published findings on TBI patients in the “ICU stratum” (admission/monitoring in an ICU) of the multicenter, longitudinal, observational “Collaborative European NeuroTrauma Effectiveness Research in TBI” (CENTER-TBI) study [10–15]. CENTER-TBI studied patients with TBI of all severities who received computed tomography (CT) of the skull within 24 h at a participating study center and consented to study participation from December 2014 to December 2017 in 65 centers from 17 European countries and Israel (13).

### Statistical analysis

For reasons of plausibility, only cases with a data entry of ≥ 50% were used for the descriptive evaluation of the TBI databank. Metric case numbers are given as mean ± standard deviation (SD) and median, categorical variables in percent. Nominal variables were compared using Fischer’s exact tests and significance was deemed to be reached at *p* < 0.05. All statistical analyses were performed using IBM SPSS Statistics (Version 25) and Graphpad PRISM (Version 7).

## Results

### Description of the TBI databank patient population

**Fig. 4:**
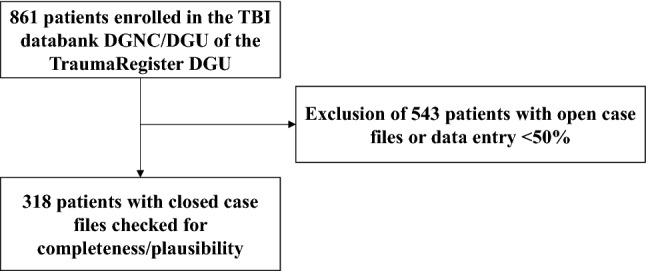
Patient population of the current TBI databank analysis

To date, 861 patients have been enrolled in the TBI databank at the nine participating hospitals, of whom 318 with completed cases and successful completeness/plausibility check could be analyzed (Fig. 4).

The mean patient age was 55 ± 23 years (median: 58 years), men and women were distributed 2.5:1 (men: *n* = 227 (71.4%); women: *n* = 91 (28.6%)). Antithrombotic medications were taken by 27.7% of patients (*n* = 86), pre-existing comorbidities were present in 46.5% (*n* = 145) and 58.2% (*n* = 171 had a severe or life-threatening systemic disease status (ASA 3–4). The most common cause of TBI was falls (55.35%; *n* = 176) and accordingly, TBI occurred most frequently in the home environment (37.1%; *n* = 118). Intubation at the scene was necessary for 44.8% (*n* = 128) of patients. According to the Glasgow Coma Scale (GCS) assessed in the shock room, severe (GCS 3–8) TBI was present in 44.7% (*n* = 122), moderate (GCS 9–12) in 10.3% (*n* = 28), and mild (GCS 13–15) in 45.1% (*n* = 123) of cases (Table 1).

**Table 1:** Demographic data and clinical findings

Variable	TBI databank
Number of patients	318
Age (mean ± SD; median)	55 ± 23; 58 years
Male sex	227/318 (71.4%)
Systemic disease according to ASA classification	
None (ASA 1)	123/294 (41.24%)
Mild (ASA 2)	87/294 (29.6%)
Severe (ASA 3)	80/294 (27.2%)
Life-threatening (ASA 4)	4/294 (1.4%)
Preexisting comorbidities (≥ 1)	145/312 (46.5%)
Antithrombotic medication	86/310 (22.4%)
Anticoagulants	32/284 (11.3%)
Platelet Inhibitors	47/252 (18.65%)
Cause of TBI	
Traffic accident (car)	18/313 (5.8%)
Traffic accident (motorcycle)	15/313 (4.8%)
Traffic accident (bicycle)	48/313 (15.3%)
Traffic accident (pedestrian)	26/313 (8.3%)
Fall (> 3 m)	35/313 (11.2%)
Fall (< 3 m)	141/313 (45.0%)
Other	30/313 (9.6%)
Place of TBI	
Domestic environment	118/318 (37.1%)
Road traffic	96/318 (30.2%)
Workplace/school	17/318 (5.3%)
Recreational	16/318 (5%)
Other	12/318 (3.7%)
Prehospital intubation	128/286 (44.8%)
GCS in the shock room (mean ± SD; median)	9.1 ± 5.4; 11
Severity of TBI	
Mild (GCS 13–15)	123/273 (45.1%)
Moderate (GCS 9–12)	28/273 (10.3%)
Severe (GCS 3–8)	122/273 (44.7%)
Injury severity according to ISS (mean ± SD; median)	23 ± 11; 22
Injury severity of the head according to AIS	
None to minor (AIS Head 0–1)	7/318 (2.2%)
Moderate to serious (AIS Head 2–3)	126/318 (39.6%)
Severe (AIS Head 4)	90/318 (28.3%)
Critical (AIS Head 5)	95/318 (29.9%)
Pupil reaction (pre-clinical)	
Normal	155/201 (77.1%)
Delayed	26/201 (12.9%)
Not reactive	20/201 (10.0%)
Pupil status	
Normal	165/221 (74.7%)
Anisocoria	33/221 (14.9%)
Dilated on both sides	23/221 (10.4%)

*TBI* traumatic brain injury, *SD* standard deviation, *ASA* American Society of Anesthesiologists, *GCS* Glasgow Outcome Sacle, *ISS* Injury Severity Score, *AIS* Abbreviated Injury Scale

In cranial computed tomography (CT) imaging, intracranial pathology was seen in 94.9% (*n* = 297), whereby the most common finding was a traumatic subarachnoid hemorrhage (tSAB; 75.6%; *n* = 220), followed by an acute subdural hematoma (aSDH; 67.3%; *n* = 191). Of note, 230 patients (73.5%) received a whole-body CT and 131 patients (42.3%) vascular imaging as part of the diagnostic workup.

While still in the shock room, 28.3% (*n* = 88) of the TBI patients underwent emergency intracranial surgery and 24.1% (*n* = 74) required invasive intracranial pressure (ICP) monitoring. During the ICU stay, further treatments for ICP lowering were performed and included surgical (17.6%; *n* = 54) and medical (16.6%; *n* = 48) interventions (Table 2).

**Table 2:** Diagnostics and therapy

Variable	TBI databank
Diagnostic imaging in the shock room	
Whole-body CT	230/313 (73.5%)
Cranial CT only	252/309 (81.6%)
Vascular imaging	131/310 (42.3%)
Cranial MRI	4/309 (1.3%)
Cranial CT findings	
Intracranial pathology present	297/313 (94.9%)
Acute subdural hematoma	191/284 (67.3%)
Epidural hematoma	50/275 (18.2%)
Traumatic subarachnoid hemorrhage	220/290 (75.9%)
Diffuse axonal damage	7/276 (2.5%)
Dislocated calvarial fracture	188/314 (59.9%)
Midline shift > 4 mm	48/310 (15.5%)
Emergency ICP monitoring	74/307 (24.1%)
Emergency intracranial surgeries	88/311 (28.3%)
Craniotomy	56/311 (18%)
Decompressive craniectomy	32/311 (10.3%)
Emergency extracranial surgeries	35/306 (11.4%)
Laparotomy	8/306 (2.6%)
Thoracotomy	4/306 (1.3%)
Stabilization of the pelvis/extremities	20/306 (6.5%)
Other	3/306 (1%)
Treatments for ICP lowering on the ICU	
EVD placement	11/304 (3.6%)
Craniotomy for hematoma evacuation	27/304 (8.9%)
Decompressive craniectomy	16/304 (5.3%)
Osmotically active substances	34/289 (11.8%)
Barbiturates	14/287(4.9%)
Overall ICP monitoring	102/306 (33.3%)
Overall intracranial surgeries	131/311 (42.1%)
Craniotomy	83/311 (26.7%)
Decompressive craniectomy	48/311 (15.4%)

*CT* computer tomography, *MRI* magnetic resonance imaging, *ICP* intracranial pressure, *ICU* intensive care unit, *EVD* external ventricular drain

The mean duration of ventilation was 7.6 ± 11.1 days (median: 1.2 days) and infectious complications such as pneumonia (in 28% of patients; *n* = 89) were common. After a mean hospital stay of 16.2 ± 13.2 days (median: 11 days), 99 patients (31.1%) could be discharged home whereas 152 patients (47.8%) were discharged to another institution. The remaining patients (*n* = 67) had died, resulting in a mortality rate of 21.1%. Interestingly, death occurred under maximum therapy in only 15 of those patients (22.4%), whereas medical reasons (23.9%; *n* = 16), family interviews (34.3%; *n* = 23) or living wills (19.4%; *n* = 13) accounted for therapy limitation in all other cases.

At transfer/discharge, 69.3% (*n* = 122) of surviving patients had a favorable treatment outcome (GOSe 5–8). At the 6-month follow-up, information on 71 patients was available, and a favorable treatment outcome was achieved in 70.4% (*n* = 50) of cases. At the 12-month follow-up, 30 patients participated, and a favorable treatment outcome was found in 90% (*n* = 27; Table 3).

**Table 3:** Clinical course and outcome

Variable	TBI databank
Complications	
Sepsis/catheter-associated infections	27/282 (9.6%)
Pneumonia	89/318 (28.0%)
Urinary tract infection	14/318 (4.4%)
Duration of ventilation (mean ± SD; median)	7.6 ± 11.1; 1.2 days
Stay in the ICU (mean ± SD; median)	10.3 ± 12.0; 5.0 days
In-hospital stay (mean ± SD; median)	16.2 ± 13.2; 12.0 days
In-hospital mortality	65/307 (21.2%)
Therapy limitation	52/307 (16.9%)
Living will	13/307 (4.2%)
Family interview	23/307 (7.5%)
Medical reasons	16/307 (5.2%)
Route of discharge/transfer	
Home	99/318 (31.1%)
Inpatient follow-up treatment	113/318 (35.5%)
Other hospital	30/318 (9.4%)
Others	9/318 (2.8%)
Outcome at discharge/transfer	
Unfavorable outcome (GOSe 1–4)	54/176 (30.7%)
Favorable outcome (GOSe 5–8)	122/176 (69.3%)
Outcome at 6-month follow-up	
Unfavorable outcome (GOSe 1–4)	21/71 (29.6%)
Favorable outcome (GOSe 5–8)	50/71 (70.4%)
Outcome at 12-month follow-up	
Unfavorable outcome (GOSe 1–4)	3/30 (10%)
Favorable outcome (GOSe 5–8)	27/30 (90%)

*SD* standard deviation, *ICU* intensive care unit, *GOSe* Glasgow Outcome Scale extended

### The TBI databank in European comparison

Compared to the 2138 TBI patients treated with intensive care in the CENTER-TBI study, similarities, and differences to results of the TBI databank can be elaborated (Table 4).

**Table 4:** The TBI databank DGNC/DGU in European comparison

Variable	TBI databank	CENTER-TBI	p value
Number of patients	318	2138	
Age (mean ± SD)	55 ± 23 years	48 ± 21 years	
Male sex	227/318 (71.4%)	1562/2138 (73.1%)	*p* = 0.5433
Severe or life-threatening pre-injury systemic disease (ASA 3–4)	210/294 (28.6%)	1818/2028 (10.4%)	*p* < 0.0001
Antithrombotic medication	86/310 (27.7%)	298/1982 (15%)	*p* < 0.0001
Blunt TBI	300/8 (94.4%)	2066/2097 (98.5%)	*p* = 0.1475
Fall as a cause of TBI	176/318 (55.35%)	839/2053 (40.9%)	*p* < 0.0001
Domestic environment as a place of TBI	118/318 (37.1%)	509/2098 (24.3%)	*p* < 0.0001
Severe TBI (GCS 3–8)	122/273 (44.7%)	961/2009 (47.8%)	*p* = 0.3335
Moderate TBI (GCS 9–12)	28/273 (10.3%)	328/2009 (16.3%)	*p* = 0.0097
Mild TBI (GCS 13–15)	123/273 (45%)	720/2009 (35.8%)	*p* = 0.0040
Severe injury to the brain/head according to the AIS (5)	95/318 (29.9%)	1014/2095 (48.4%)	*p* < 0.0001
Pupils unreactive	20/201 (9.95%)	380/2016 (18.85%)	*p* = 0.0014
Midline shift > 4 mm on brain CT imaging	48/310 (15.5%)	577/1998 (28.9%)	*p* < 0.0001
Diffuse axonal injury on brain CT imaging	7/276 (2.5%)	341/1531 (18.2%)	*p* < 0.0001
Invasive ICP monitoring performed	102/306 (33.3%)	921/2113 (43.6%)	*p* = 0.0007
Craniotomy performed	83/311 (26.7%)	197/2115 (9.3%)	*p* < 0.001
Decompressive craniectomy performed	48/311 (15.4%)	212/2115 (10%)	*p* = 0056
Pneumonia as an infectious complication	89/318 (28%)	280/2090 (13.4%)	*p* < 0.0001
Sepsis as an infectious complication	27/292 (9.6%)	36/1464 (2.5%)	*p* < 0.0001
In-hospital mortality	67/318 (21.1%)	318/1918 (16.6%)	*p* = 0.0542
Limitation of therapy due to medical reasons	16/307 (5.2%)	181/1918 (9.4%)	*p* = 0.0129
Limitation of therapy due to a living will	13/307 (4.2%)	31/1918 (1.6%)	*p* = 0.0061
Limitation of therapy due to the family interview	23/307 (7.5%)	32/1918 (1.7%)	*p* < 0.0001
Length of in-hospital stay (mean ± SD)	16.2 ± 13.2 days	25.3 ± 33.8 days	
Discharge to an inpatient rehabilitation facility	113/318 (35.5%)	422/1600 (26.4%)	*p* = 0.0013
Favorable outcome (GOSe 5–8) after 6 months	50/67 (75%)	1051/1846 (57%)	0.0037

*CENTER-TBI* Collaborative European NeuroTrauma Effectiveness Research in TBI, *SD* standard deviation, *ASA* American Society of Anesthesiologists, *TBI* traumatic brain injury, *GCS* Glasgow Coma Scale, *AIS* Abbreviated Injury Scale, *CT* computer tomography, *ICP* intracranial pressure, *GOSe* Glasgow Outcome Scale extended

TBI with ICU admission in Germany as in Europe is frequently severe (GCS 3–8; 44.7% vs. 47.8%; *p* = 0.3335), affects predominantly men (71.4% vs. 73.1%; *p* = 0.5433), and mostly results from blunt injuries (97.4% vs. 98.5%; *p* = 0.1475). However, TBI patients treated in an ICU in Germany seem to be older (55 ± 23 years vs. 48 ± 21 years), more pre-diseased (ASA 3–4 in 28.6% vs. 10.4%; *p* < 0.0001), more commonly treated with antithrombotic drugs (27.7% vs. 15%; *p* < 0.0001) and more likely to suffer a fall (55.35% vs. 40.9%; *p* < 0.0001) in the home environment (37.1% vs. 24.3%; *p* < 0.0001) compared to their European neighbors.

In contrast, clinical (AIS Head 5 in 29.9% vs. 48.4%; *p* < 0.0001 or pupils unreactive in 9.95% vs. 18.85%; *p* = 0.0014), as well as image morphological injury severity (midline shift > 4 mm in 15.5% vs. 28.9%; *p* < 0.0001 or diffuse axonal injury in 2.5% vs. 18.2%; *p* < 0.0001), might be milder in Germany.

Correspondingly, invasive ICP monitoring is less often performed in German TBI patients than in CENTER-TBI ICU patients (33.3% vs. 43.6%; *p* = 0.0007). However, craniotomies (26.7% vs. 9.3%; *p* < 0.001) and decompressive craniectomies (15.4% vs. 10%; *p* = 0056) after TBI are more common in Germany than in Europe.

Of note, infectious complications of TBI patients seem to occur more often in Germany (pneumonia in 28% vs. 13.4%; *p* < 0.0001 and sepsis in 9.6% vs. 2.5%; *p* < 0.0001) and in-hospital mortality after TBI is slightly increased compared to the CENTER-TBI ICU patients (21.1% vs. 16.6%; *p* = 0.0542). In terms of therapy limitation after TBI, medical reasons are stated less often in Germany than in Europe (5.2% vs. 9.4%; *p* = 0.0129), whereas living wills (4.2% vs. 1.6%; *p* = 0.0061) and family interviews (7.5% vs. 1.7%; *p* < 0.0001) are considered more often.

Nevertheless, the duration of treatment in the acute hospital is shorter in the TBI databank (16.2 ± 13.2 days vs. 25.3 ± 33.8 days) and patients are more often discharged to inpatient rehabilitation facilities (35.5% vs. 26.4%; *p* = 0.0013). Moreover, despite demographic and epidemiological differences, the treatment outcome after six months seems better in Germany compared to its European neighbors (GOSe 5–8 in 75% vs. 57%; *p* = 0.0037).

## Discussion

Through close interdisciplinary collaboration between neurosurgeons and traumatologists, support and funding of their national societies and effective utilization of the TR-DGU as a preexisting data collection structure, the German national TBI databank DGNC/DGU could be established within only five years. The current analysis of its first 318 TBI patients prospectively enrolled in the ICUs of nine participating hospitals enables insights into the epidemiology, treatment, and outcome of TBI in Germany today. Key findings are a progressive demographic change towards older and frailer TBI patients, high rates of mild TBI admissions to the ICU but also intracranial traumatic pathologies and surgeries, still a relevant number of in-hospital deaths and mainly favorable long-term outcome in TBI survivors.

### Data protection and ethical aspects of the TBI databank

The high data protection requirements of the TBI databank presented a challenge during its implementation. According to the currently valid European Data Protection Regulation (EU-DSGVO) and the German Federal Data Protection Act (BDSG) of May 25, 2018, patients must give informed consent to use their data for scientific purposes, even in the case of pseudonymized registries. Accordingly, the AUC prepared a legally compliant information notice and a consent form for data transfer, quality assurance, and research purposes for patients and legal guardians, which are necessary for the enrollment of patients in the existing TR-DGU and the TBI databank. However, obtaining informed consent for the TBI databank so far proven particularly difficult for the timely inclusion of TBI patients with impaired consciousness.

The professional ethics and legal advice from the responsible ethics committees required for the local establishment of the TBI databank also posed a hurdle. The AUC only approves hospitals for the TBI databank if a local, positive ethics vote is available. Furthermore, a contractual addendum to the TR-DGU contract with the AUC is required for each hospital to use the TBI databank's data collection platform. These time-consuming formal processes reduced the number of hospitals that could so far be initiated for participation in the TBI databank from 18 which have already started the process to currently nine.

### TBI patients receiving intensive care in Germany

Data on TBI patients receiving intensive care collected in the TBI databank confirm the continuing demographic and epidemiological shift toward older populations with relevant comorbidities who frequently fall in the home environment [2, 4, 16, 17]. However, our current analysis from the TBI databank shows no relevant change compared with a cohort of TBI patients analyzed from the TR-DGU between 2013 and 2017 in median patient age (58 years vs. 60 years), sex distribution (71% vs. 68% men) or most common cause of TBI (falls in 55% vs. 39%) [6]. Of note, only patients with moderate-to-severe TBI were included in this latter analysis. For the first time, therefore, the TBI databank depicts the reality of TBI care in German ICUs, revealing that patients are treated or monitored there with mild TBI in 45% of cases. Similar but still lower figures have already been demonstrated in prospective observational studies for Europe (35.8%) and the USA (20%) [15, 18]. Hypotheses for this include older patient age, relevant concomitant diseases, and use of antithrombotic medications with the risk of intracranial hemorrhage progression—the present data confirm these.

Furthermore, injury severity with a median Injury Severity Score (ISS) of 24 (ISS ≥ 16 defines a polytrauma [19]) and the rate of extracranial emergency surgeries of 11.4% in the TBI databank also suggest that extracranial injuries may somewhat have been the reason for intensive care treatment of TBI patients. However, to date, there is no evidence that intensive care monitoring of patients with mild TBI improves treatment outcomes, and thus reducing the number of such patients treated in the ICUs could result in cost savings in acute TBI care in Germany [18]. In this context, it is striking that in the TBI databank, compared with the analysis of moderate-to-severe TBI in the TR-DGU, intracranial emergency surgery was documented in more than three times as many patients (66% vs. 18%). A fact that might be related to the overproportioning of neurosurgical vs. traumatological hospitals (7:2) enrolling patients in the TBI databank so far.

Notably, the current data from the TBI databank demonstrates that the mortality of TBI remains relevant at 21% and underscores the statistics on deaths of all patients treated as full inpatients in Germany for head injury (ICD-10: S00-S09), also collected in 2019; these nearly doubled from 3468 in 2000 to 7042 [1]. What is more, the TBI databank shows for the first time for Germany that this mortality often seems to be caused by deliberate therapy limitation (78% of cases), which is in line with a trend in Europe as well [20]. As a novelty, the current data also provide insight into the longer-term treatment outcome of TBI patients, which in some cases continues to improve after hospital discharge. Considering the high rate of direct transfer to specialized inpatient follow-up treatment in Germany (35%) revealed by the TBI databank, this may allow conclusions about the importance of rehabilitation after TBI in the future.

### Differences between the TBI databank and CENTER-TBI patients

One strength of the data set of the newly established TBI databank is its harmonization with other international data collection structures. In Europe, however, so far only a few comparable projects exist The Danish “Head Trauma Database” which collects data from two national neurorehabilitation hospitals and the Swedish “National Quality Registry for TBI” which enrolls TBI patients from the Uppsala region seems to be currently ongoing [21, 22]. Other prospective data collections such as the Italian “National Registry of Severe Acquired Brain Injury” which recruited 1469 severe brain injury patients from 2005 to 2007 or the Dutch “Prehospital Registry on Outcome, Treatment and Epidemiology of Cerebral Trauma” (BRAIN-PROTECT) study which focused on the prehospital, helicopter-based severe TBI management and published its results on 2589 patients in 2020 have already been terminated [23, 24]. Of note, none of those data collection structures has a similarly high number of variables, includes a similar follow-up, or uses a harmonized data set. Thus, for a comparison of TBI data collected in Germany to its European neighbors, the longitudinal observational CENTER-TBI study with > 2500 collected variables and a 12-month follow-up was better suited. Nevertheless, because differences exist in the inclusion/exclusion criteria of both data sets (CENTER-TBI excluded patients with severe preexisting neurological disorders which the TBI databank doesn’t) and because a fraction of the CENTER-TBI patients were enrolled in Germany, this analysis must be interpreted with caution.

While some similarities seem to exist, the TBI patients treated in ICUs in the TBI databank since 2019 were already older, frailer, fell more commonly at home and had a higher rate of antithrombotic medication than their counterparts in the CENTER-TBI study who were enrolled between 2014 and 2017. These findings might be related to demographic differences between Germany and its European neighbors, but giving the chronological order of both data collections, they also suggest a steep continuation of the well-documented demographic change in the European TBI population [2].

Still, we found that the overall injury severity was lower in the German TBI patients, which corresponded well to e.g., the reduced rate of invasive ICP monitoring documented in the TBI databank. However, craniotomies and decompressive craniectomies after TBI were potentially more common in Germany than in Europe, and therapy seemed to be less often limited due to medical reasons, which, taken together, could be interpreted as a trend toward overtreatment [25]. Similarly, the already high rate of mild TBI admissions to ICUs in the CENTER-TBI study was even exceeded in the TBI databank, indicating a potential trend to over surveillance in Germany.

Of note, infectious complications of TBI patients seem occur more often in Germany which may also reflect the older and more pre-diseased patient population [26]. These findings could also partly explain the slightly increased mortality after TBI in Germany and thus underline the relevance of age and previous diseases as prognostically unfavorable factors [27, 28].

On the other hand, the shorter duration of treatment in the acute hospital suspected in Germany indicates a possibly special care structure, characterized by a high rate of early transfer to inpatient rehabilitation facilities [29]. This in turn could have affected the improved 6-monhts outcome in the TBI databank compared to the CENTER-TBI study, potentially underlining among other things, the relevance of early rehabilitation after TBI [30].

### The future of the TBI databank and its limitations

To achieve a comprehensive registration of TBI patients in Germany, the activation of as many hospitals for participation as possible will be necessary in the future. Then the potential of the TBI databank is great to represent the national care reality of patients with TBI and intensive care treatment and to review common therapeutic concepts and guidelines. For participating hospitals, the AUC will also provide internal benchmarking of TBI care; national and even international benchmarking are possible in the future. However, the TBI databank remains limited in its breadth by its link to the TR-DGU, whose inclusion criteria make it impossible to map the care of TBI patients in emergency departments or regular wards. Whether expanding the TBI databank to include these care pathways is conceivable as acceptance increases remains to be seen. In addition, patients who died before hospital admission have not yet been recorded in the TR-DGU, which could further distort the comprehensive validity of the TBI databank. Due to the high data protection requirements with necessary consent for participation in data collection, there is also the possibility that, for example, unconscious patients could be underrepresented in the TBI databank. And finally, the comprehensive dataset of the TBI databank remains, on the one hand, one of its distinguishing features and, on the other hand, a possible limitation since it could also lead to low data entry rates and might require further adjustments and reductions in the future.

## Data Availability

The data that support the findings of this study are available from the corresponding author, AY, upon reasonable request.
